# RNA-Sequencing of *Heterorhabditis* nematodes to identify factors involved in symbiosis with *Photorhabdus* bacteria

**DOI:** 10.1186/s12864-022-08952-4

**Published:** 2022-11-07

**Authors:** Chaitra G. Bhat, Roli Budhwar, Jeffrey Godwin, Adler R. Dillman, Uma Rao, Vishal S. Somvanshi

**Affiliations:** 1 Division of Nematology, ICAR-Indian Agricultural Research Institute, New Delhi, Delhi, 110012 India; 2 Bionivid Technology Private Limited, 209, 4th Cross Rd., B. Channasandra, Kasturi Nagar, Bengaluru, Karnataka 560043 India; 3 Department of Nematology, University of California, Riverside, 92521 USA

**Keywords:** Biofilm, Early-adult stage, *Heterorhabditis*, Immunity, *Photorhabdus*, RNA-sequencing, Symbiosis, Transcriptome

## Abstract

**Background:**

Nematodes are a major group of soil inhabiting organisms. *Heterorhabditis* nematodes are insect-pathogenic nematodes and live in a close symbiotic association with *Photorhabdus* bacteria. *Heterorhabditis-Photorhabdus* pair offers a powerful and genetically tractable model to study animal-microbe symbiosis. It is possible to generate symbiont bacteria free (axenic) stages in *Heterorhabditis*. Here, we compared the transcriptome of symbiotic early-adult stage *Heterorhabditis* nematodes with axenic early-adult nematodes to determine the nematode genes and pathways involved in symbiosis with *Photorhabdus* bacteria.

**Results:**

A de-novo reference transcriptome assembly of 95.7 Mb was created for *H. bacteriophora* by using all the reads. The assembly contained 46,599 transcripts with N50 value of 2,681 bp and the average transcript length was 2,054 bp. The differentially expressed transcripts were identified by mapping reads from symbiotic and axenic nematodes to the reference assembly. A total of 754 differentially expressed transcripts were identified in symbiotic nematodes as compared to the axenic nematodes. The ribosomal pathway was identified as the most affected among the differentially expressed transcripts. Additionally, 12,151 transcripts were unique to symbiotic nematodes. Endocytosis, cAMP signalling and focal adhesion were the top three enriched pathways in symbiotic nematodes, while a large number of transcripts coding for various responses against bacteria, such as bacterial recognition, canonical immune signalling pathways, and antimicrobial effectors could also be identified.

**Conclusions:**

The symbiotic *Heterorhabditis* nematodes respond to the presence of symbiotic bacteria by expressing various transcripts involved in a multi-layered immune response which might represent non-systemic and evolved localized responses to maintain mutualistic bacteria at non-threatening levels. Subject to further functional validation of the identified transcripts, our findings suggest that *Heterorhabditis* nematode immune system plays a critical role in maintenance of symbiosis with *Photorhabdus* bacteria.

**Supplementary Information:**

The online version contains supplementary material available at 10.1186/s12864-022-08952-4.

## Background

Animal-microbe interactions range from facultative to obligate associations, and also from pathogenesis to mutualistic relationships. The mutualistic associations between animals and microbes are manifested as simple mono-specific associations (for e.g., nematodes-bacteria symbiosis), simple consortia (2–25 bacterial species in an animal, for e.g., leach gut consortium, insect gut consortium), and highly complex consortia (for e.g., vertebrate guts colonized by > 10^2^ to 10^3^ species of bacteria) [1]. It is well established that symbionts can affect the physiology, nutrition, metabolism, immunity, behaviour, growth and development of an animal host, and can provide protection to the host [2]. Insect-pathogenic nematodes of the genus *Steinernema* and *Heterorhabditis* live in symbiosis with the gram-negative bacteria of the genus *Xenorhabdus* and *Photorhabdus,* respectively. The nematodes carry the bacteria to newer insect hosts is soil, whereas the bacteria kill the insects and facilitate nematode growth and multiplication inside the insect cadaver. These nematodes have co-evolved with their bacterial symbionts for millions of years and form specific symbiotic relationships with their primary symbiont. Recent evidence suggests that *Steinernema*-bacteria associations may be more complex [3], however, the insect parasitic nematode *Heterorhabditis* maintains a mono-specific symbiotic relationship with *Photorhabdus* and offers a tractable genetic model for the study of animal-microbe relationships [4, 5].

**Fig. 1:**
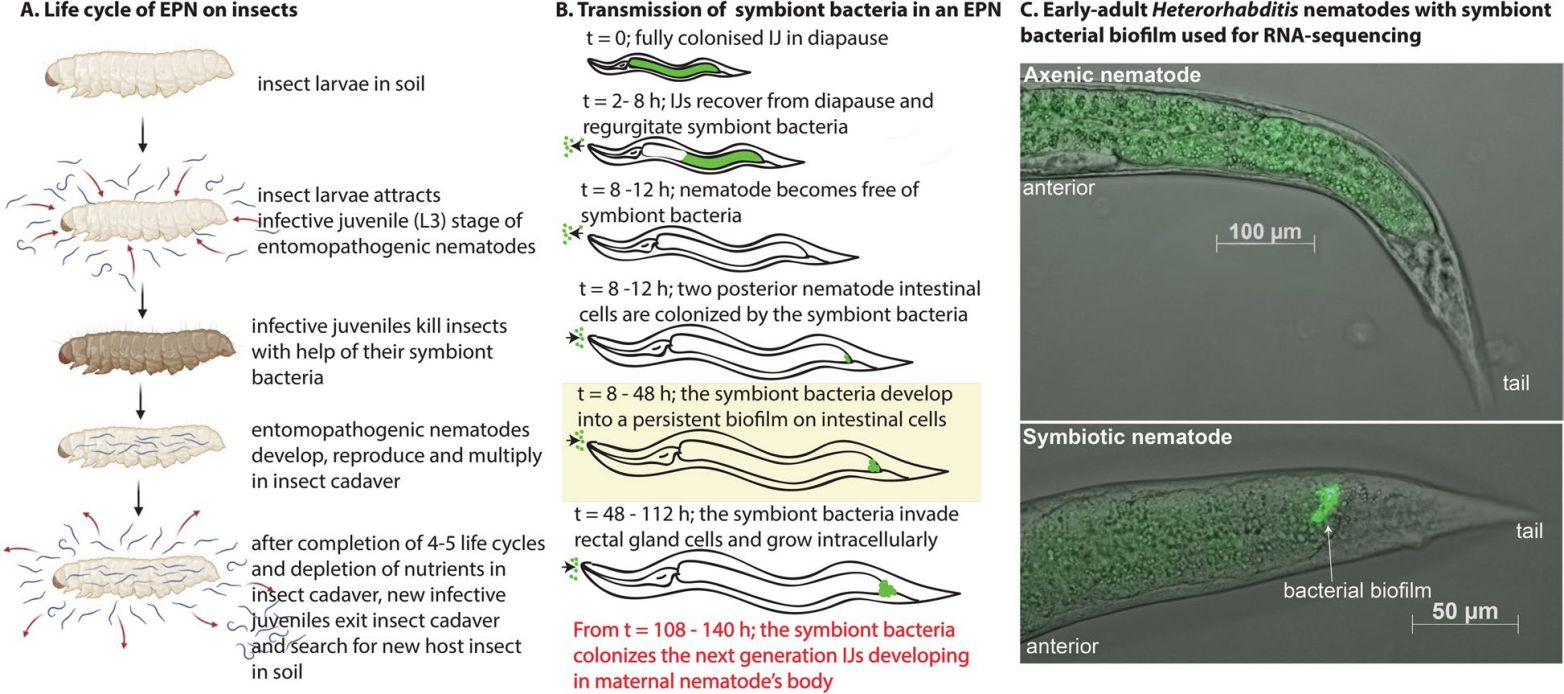
**A** The general life cycle of the entomopathogenic nematode *Heterorhabditis* on an insect (made with help of BioRender). **B** The diagrammatic representation of initial stages of the maternal transmission of the symbiont bacteria. When the IJs exit diapause, they regurgitate the symbiont bacteria and become completely bacteria free before reassociating with their symbiont bacteria later on during the development. The reassociation of symbiont bacteria results in formation of a persistent symbiont bacterial biofilm in the posterior intestinal cells on the nematode. The green colour shows bacteria, arrow indicates direction of bacterial movement, and the yellow shaded box indicates early-adult stage of nematode. **C** Early-adult stage of symbiotic and axenic *Heterorhabditis bacteriophora* used for RNA-sequencing. The image was created by overlapping DIC and fluorescence images of axenic early-adult devoid of bacterial biofilm and the bacterial biofilm in the posterior intestine of early-adult stage of symbiotic nematode

The third stage of *Heterorhabditis* nematodes, which is also the infective stage (also known as infective juveniles (IJs)) is found freely in soil. The IJs carry the symbiont *Photorhabdus* bacteria in their gut. Once the IJs find an insect host in soil, they enter the insect body through natural openings or by direct penetration. Upon reaching the insect haemocoel, the IJs regurgitate the symbiont bacteria into the insect hemocoel (Fig. 1 A), where these bacteria multiply and cause rapid insect death by septicaemia or also by producing numerous protein toxins and secondary metabolites. Later, the nematodes resume their development and reproduce in the insect cadaver, complete 3–5 generations and when the cadaver is depleted of food, the *Heterorhabditis* IJs emerge from the cadavers in high numbers and disperse in soil in search of a new host-insect. The bacteria are transmitted to the offspring nematodes maternally in a sophisticated and developmentally controlled manner (Fig. 1 B) [6]. The IJs regurgitate the symbiont bacteria and become completely free of the bacteria in the insect hemocoel before they resume development to fourth stage juvenile in the next hours. Once the nematode development is resumed, the nematodes start feeding on *Photorhabdus* and growing. A sub-population of *Photorhabdus*, known as the ‘M-form' attaches to posterior nematode intestinal cells INT9L and INT9R and makes a persistent biofilm (Fig. 1 B) [6]. Early-adult stage of *Heterorhabditis* nematode (36–40 h after IJ resumes development) marks the beginning of symbiotic transmission as the *Photorhabdus* symbiont attach and form a robust and persistent biofilm at the posterior nematode intestinal cells INT9L and INT9R [6]. Hence, this stage was used for comparing the transcriptomes of symbiotic and axenic nematodes. As the hermaphrodite nematodes grow and with passage of time, the bacterial cells of the biofilm invade the nematode rectal gland cells and are internalised in the vacuoles [6]. Later, these vacuoles rupture and the *Photorhabdus* cells contained in these vacuoles colonise the freshly hatched nematode juveniles developing inside their mother’s body through endotokia matricida, thereby completing the symbiont transmission from the mother to the offspring (Fig. 1 B) [6]. Both the morphology and physiology of the symbiont colonization sites in the nematode intestine, and the developmental progression of this symbiotic relationship has been elucidated [6]. Availability of sequenced genomes of both, the bacteria and the nematode has greatly facilitated functional genomics and genetic interrogations for the *Heterorhabditis*-*Photorhabdus* system [7–11]. This has also enabled identification of some of the bacterial genes and processes necessary for symbiotic colonization of the nematodes [12–15].

In spite of all the new developments in the understanding of the *Heterorhabditis*-*Photorhabdus* symbiosis system, the host nematode factors needed for symbiosis or host specificity are largely unknown. A deeper understanding of this symbiosis, including the relative roles played by the microbe versus those by the host nematodes may yield insights into fundamental processes underlying the ubiquitous association of microbes with the animals.

In the present study, a comparative transcriptomic analysis of the early-adult stage of symbiotic and axenic *H. bacteriophora* was carried out to understand the molecular processes and pathways active during the initial stage of nematode-bacteria symbiosis.

## Results

### RNA-Sequencing of *Heterorhabditis* nematodes at symbiont biofilm formation stage

Early-adult stage of *Heterorhabditis* marks the beginning of symbiotic transmission as the *Photorhabdus* symbiont attaches and forms a biofilm at the posterior intestinal cells of the nematode (Fig. 1 B). Hence, the early-adult hermaphrodite nematodes were visualized under the microscope and the presence of symbiont bacterial biofilm in the posterior intestinal cells of the symbiotic (S) nematodes was confirmed, whereas no biofilm was found in the axenic (A) nematodes at the same developmental stage (Fig. 1 C). Total RNA was extracted from each of 100 μl pellet of symbiotic and axenic early-adults of *H. bacteriophora* separately, checked for quality and used for library preparation and sequenced as per details provided in the methods section. Approximately 27 to 40 million raw reads were generated per sample for the three independent biological replicates for each of axenic and symbiotic nematodes (Table 1). Read quality filtering by fastp tool resulted in selection of > 94% paired-end high quality (HQ) reads per sample. A total of 26.28 to 40.58 million HQ reads were obtained for the three replicates of axenic nematodes, while 31.91 to 39.60 million HQ reads were obtained for the three replicates of symbiotic nematodes, respectively (Table 1). Reads from the three biological replicates showed correlation coefficients from 0.83 to 0.95 and 0.77 to 0.91 for axenic and symbiotic nematodes, respectively (supplementary table S1). Raw sequence data has been deposited in Sequence Read Archive, with accession numbers SRR14162364, SRR14162365, SRR14162366, SRR14162367, SRR14162368, and SRR14162369. Bio-sample accession number is SAMN18646821, and Bio-project accession number is PRJNA720314.

**Table 1:** Raw and filtered read statistics for the RNA-Seq experiment

Sample	Raw reads			Filtered reads			
	Total reads	Total bases (Giga bases)	GC %	Total reads	Total bases (Giga bases)	GC %	% HQ Reads
Axenic (Rep 1)	27,337,042 For:13,668,521 Rev:13,668,521	4.10	44.02	26,288,598 For: 13,144,299 Rev: 13,144,299	3.56	43.32	96.16
Axenic (Rep 2)	29,222,012 For:14,622,006 Rev:14,622,006	4.38	43.99	27,979,572 For: 13,989,786 Rev:13,989,786	3.87	43.33	95.74
Axenic (Rep 3)	41,116,354 For:20,558,177 Rev:20,558,177	6.16	41.45	40,583,640 For: 20,291,820 Rev:20,291,820	6.06	41.44	98.71
Symbiotic (Rep 1)	33,448,662 For: 16,724,331 Rev:16,724,331	5.01	42.75	31,915,618 For: 15,957,809 Rev:15,957,809	4.38	41.87	95.42
Symbiotic (Rep 2)	36,100,070 For: 18,050,035 Rev:18,050,035	5.41	44.26	34,093,176 For: 17,046,588 Rev:17,046,588	4.59	43.41	94.44
Symbiotic (Rep 3)	40,100,056 For: 20,050,028 Rev:20,050,028	6.01	40.99	39,603,638 For: 19,801,819 Rev:19,801,819	5.90	40.96	98.76

### Transcriptome assembly, completeness and annotation

The HQ sequence data from all the samples was used to generate a de-novo reference assembly of 95.7 Mb using Trinity assembler. Assembly was improved by amelioration, removing the duplicates by CD_HIT_EST resulting in the assembly of 46,599 transcripts (supplementary information S2). N50 value of the assembly was 2,681 bp, and the average transcript length was 2,054 bp. The statistical details for the assembly are provided in Table 2. Transcriptome assembly completeness was assessed using BUSCO against eukaryota_odb10.2019–11-20 (255 genes) database, which showed the presence of 95.3% complete (C) including 29.40% complete and single copy (S) and 65.90% Complete and duplicated BUSCOs (D), 2% fragmented (F) and 2.7% missing (M) genes (Table 2). A comparison to nematoda_odb10.2019–11-20 (3,131 genes) database found 93.6% BUSCOs as complete (C), 1.6% as fragmented (F) and 4.8% as missing (M) (Table 2). Approximately 97% of the reads across the samples mapped backed to our transcriptome assembly.

**Table 2:** Assembly statistics and completeness assessment (by BUSCO against Eukaryota and Nematoda databases) of the *de-novo H. bacteriophora* transcriptome assembly

Assembly statistics		
Number of transcripts	46,599	
Total bases (bp)	95,741,839 (95.7 Mb)	
Minimum sequence length (bp)	501	
Maximum sequence length (bp)	19,436	
Average sequence length (bp)	2,054.59	
N50 value (bp)	2,681	
(G + C)s	38.11%	
**Assembly completeness assessment using BUSCO**		
*Against eukaryota_odb10.2019–11-20* (Total BUSCO groups = 255)		
Complete BUSCOs (C)	243	95.30%
Complete and single-copy BUSCOs (S)	75	29.40%
Complete and duplicated BUSCOs (D)	168	65.90%
Fragmented BUSCOs (F)	5	2%
Missing BUSCOs (M)	7	2.70%
*Against nematoda_odb10.2019–11-20* (Total BUSCO groups = 3,131)		
Complete BUSCOs (C)	2,930	93.60%
Complete and single-copy BUSCOs (S)	1,109	35.40%
Complete and duplicated BUSCOs (D)	1,821	58.20%
Fragmented BUSCOs (F)	50	1.60%
Missing BUSCOs (M)	151	4.80%

Of the total 46,599 transcripts, 33,762 (72.45%) could be annotated using blastx against Uniprot90 / SwissProt database and Trinotate tool. Ref-Seq analysis revealed that nematode genes were the top hits for 17,952 of the transcripts. Functional characterization of transcripts using Gene Ontology (GO) resulted in 33,760 annotated transcripts. The top ten GO terms enriched in the early-adult *H. bacteriophora* transcriptome under each category of molecular function, biological processes and cellular components are provided in supplementary figure S1. Positive regulation of transcription by RNA polymerase II (GO:0045944), multicellular organism development (GO:0007275) and cell differentiation (GO:0030154) were the top three GO terms under biological process category with 936, 921 and 725 transcripts, respectively. Nucleus (GO:0005634), cytoplasm (GO:0005737) and integral component of membrane (GO:0016021) were the most enriched GO terms under cellular component category with 7,493, 7,220 and 6,352 transcripts, respectively. Under the molecular function category, ATP binding (GO:0005524), metal ion binding (GO:0046872), and DNA binding (GO:0003677) were the top three terms with 4,538, 4,397 and 1,689 transcripts, respectively. Functional annotation by KAAS database resulted in the annotation of 19,864 transcripts grouping into 297 pathways, out of which Thermogenesis (ko04714), Endocytosis (ko04144), and Protein processing in Endoplasmic Reticulum (ko04141) pathways were the most abundant (supplementary information S3).

### Differentially expressed transcripts

The relative expression of transcripts in all the three replicates of symbiotic and axenic early-adults of *Heterorhabditis* based on FPKM values is provided in supplementary figure S2. The transcripts that were differentially expressed in the symbiotic nematodes as compared to axenic nematodes were identified by using DESeq package. A total of 754 transcripts (1.62% of the total) were differentially expressed in symbiotic nematodes as compared to axenic nematodes. The number of down-regulated transcripts was 547, whereas 207 transcripts were up-regulated in the symbiotic nematodes (supplementary figure S2). A list of top ten up- and down-regulated transcripts is provided in Table 3. Interestingly, 12,151 transcripts (26.05% of total) were exclusive to symbiotic nematodes, whereas 20 transcripts were found only in axenic nematodes.

**Table 3:** Topmost up- and down-regulated genes (with annotation) in symbiotic nematodes as compared to axenic nematodes

	**Transcript ID**	**Fold change**	**Gene**	**Uniprot identifier**	**Protein**
	**Down-regulated transcripts**				
1.	TRINITY_DN6295_c0_g10_i1--LEN=742	-11.5	*COII*	P29870	Cytochrome c oxidase subunit 2
2.	TRINITY_DN3587_c0_g1_i1--LEN=1046	-10.55	*RPLP0*	Q95140	60S acidic ribosomal protein P0
3.	TRINITY_DN7104_c0_g6_i1--LEN=722	-10.51	*TEF-1*	P28295	Elongation factor 1-alpha
4.	TRINITY_DN7997_c0_g4_i3--LEN=1041	-10.47	*RpL8*	P41569	60S ribosomal protein L8
5.	TRINITY_DN6295_c0_g4_i1--LEN=1062	-10.41	*MT-CYB *	Q9XNX3	Cytochrome b
6.	TRINITY_DN6392_c1_g2_i4--LEN=1245	-10.31	*RPL11B *	P42794	60S ribosomal protein L11-2
7.	TRINITY_DN5202_c0_g4_i1--LEN=677	-10.16	*RPL13A*	Q3SZ90	60S ribosomal protein L13a
8.	TRINITY_DN6050_c0_g4_i1--LEN=522	-10.09	*RPL23AA *	Q8LD46	60S ribosomal protein L23a-1
9.	TRINITY_DN7721_c0_g10_i1--LEN=507	-10.08	*RPS19S*	P39698	40S ribosomal protein S19S
10.	TRINITY_DN3803_c0_g2_i1--LEN=1053	-10.06	*-*	Q93134	Guanine nucleotide-binding protein subunit beta-2-like 1
	**Up-regulated transcripts**				
1.	TRINITY_DN821_c0_g1_i1--LEN=940	3.93	*col-19 *	P18835	Cuticle collagen 19
2.	TRINITY_DN4551_c0_g2_i3--LEN=723	3.32	*gsnl-1 *	A8XV95	Gelsolin-like protein 1
3.	TRINITY_DN6239_c0_g7_i1--LEN=5066	3.23	*vit-6 *	P18948	Vitellogenin-6
4.	TRINITY_DN5914_c2_g3_i1--LEN=1072	3.10	*col-34 *	P34687	Cuticle collagen 34
5.	TRINITY_DN4902_c0_g1_i1--LEN=2737	3.07	*EHMT1 *	Q9H9B1	Histone-lysine N-methyltransferase EHMT1
6.	TRINITY_DN6718_c0_g2_i7--LEN=855	2.96	*TUBA2*	P33624	Tubulin alpha-2 chain
7.	TRINITY_DN1416_c0_g2_i1--LEN=1071	2.89	*clec-87 *	A8WUV1	C-type lectin domain-containing protein 87
8.	TRINITY_DN2156_c0_g1_i1--LEN=1108	2.86	*moe-3 *	Q9XV46	CCCH-type zinc finger protein moe-3
9.	TRINITY_DN992_c0_g1_i2--LEN=1401	2.81	*tbx-8 *	Q22292	T-box transcription factor tbx-8
10.	TRINITY_DN39_c0_g1_i2--LEN=1805	2.76	*APX1 *	Q05431	L-ascorbate peroxidase 1, cytosolic

**Fig. 2:**
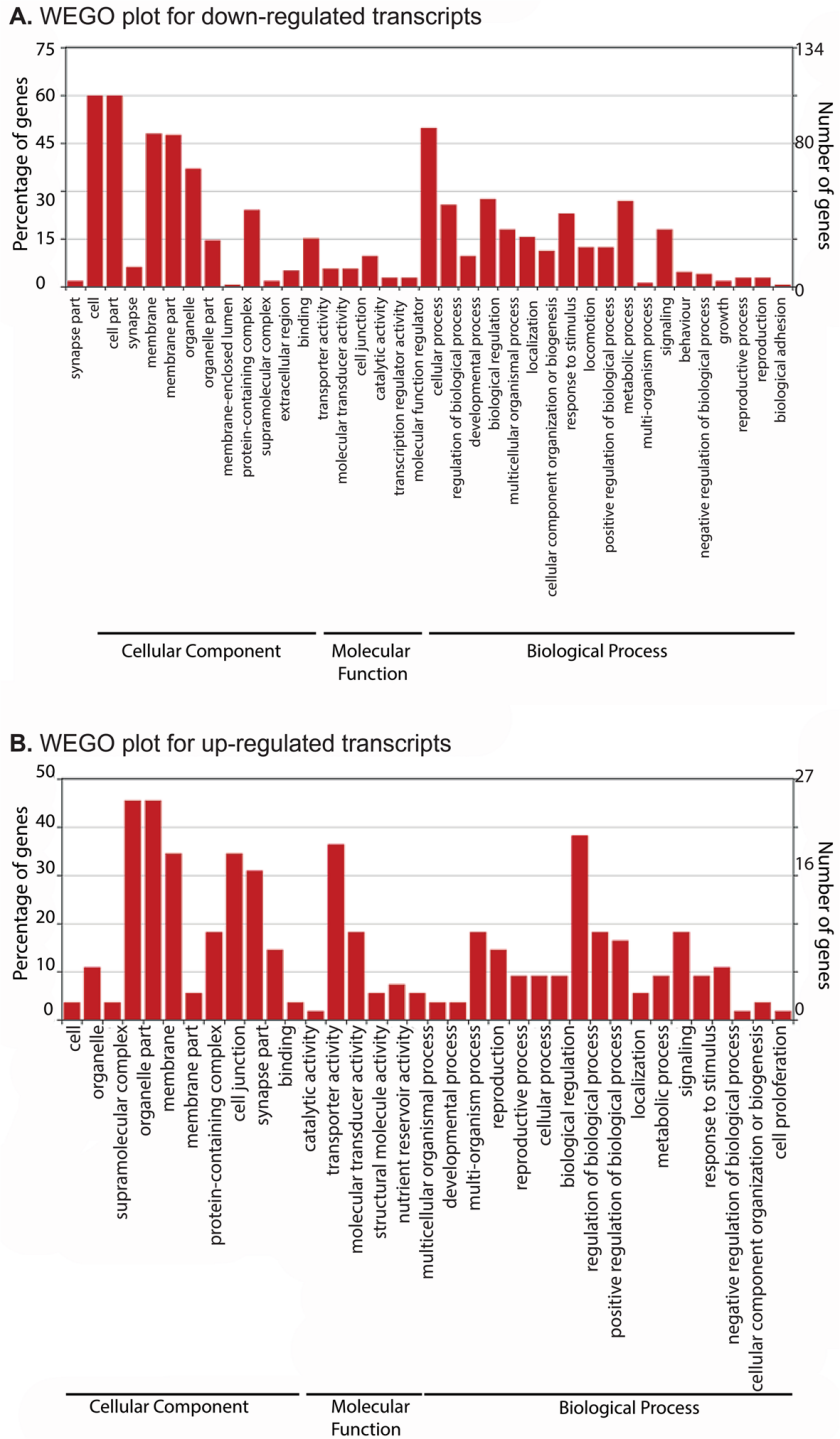
Gene Ontology (GO) annotation of differentially expressed transcripts identified in RNA-seq experiment. **A**. up-regulated transcripts **B**. down-regulated transcripts. WEGO (Web Gene Ontology Annotation Plot) tool was used for visualizing, comparing and plotting GO annotation results

**Fig. 3:**
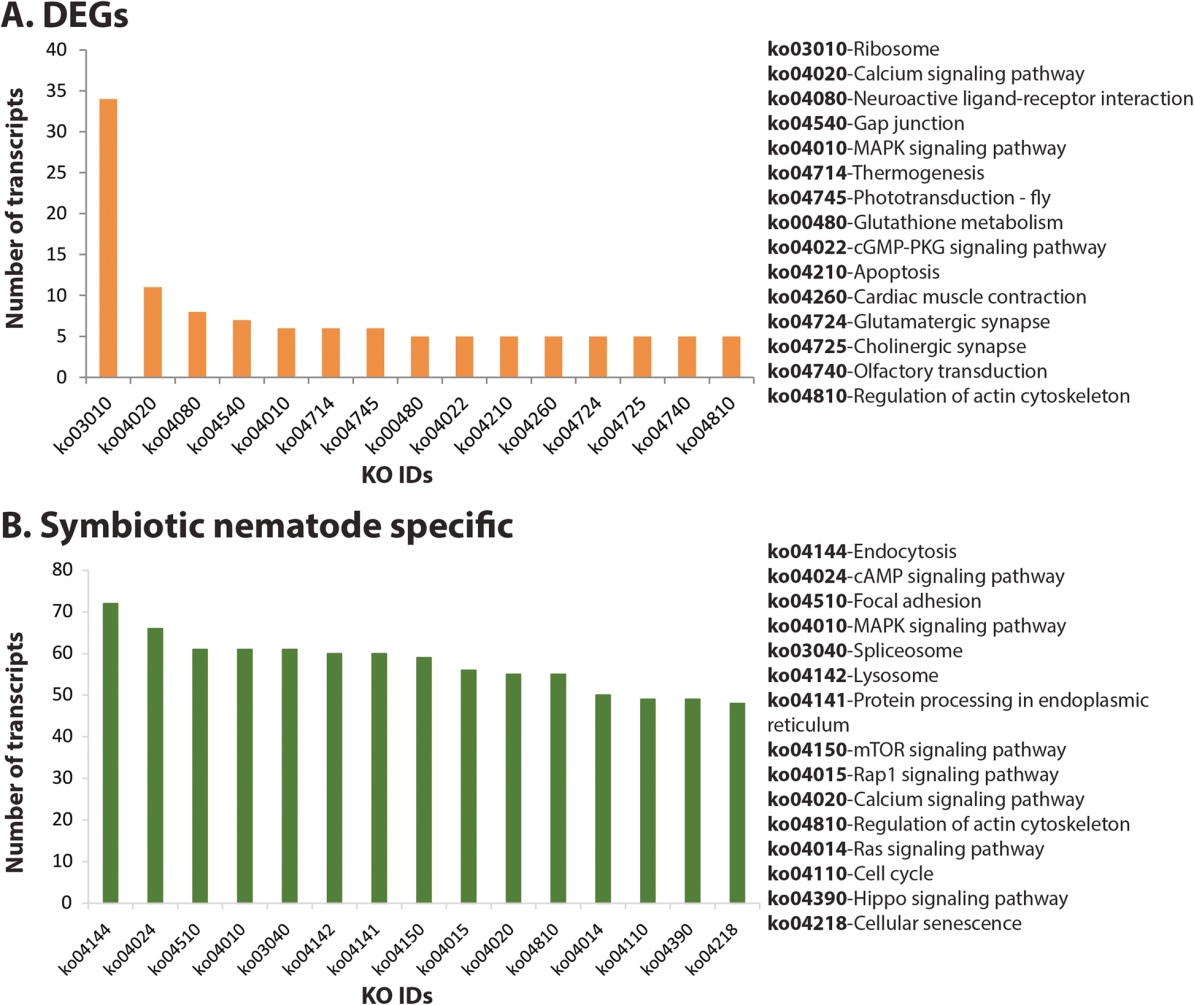
Pathways enriched in identified transcripts. **A**. KEGG pathway annotations of differentially expressed transcripts. **B**. KEGG pathway annotations of symbiotic-nematode specific transcripts. KEGG Automatic Annotation Server (KAAS) (https://www.genome.jp/kegg/kaas/) was used for pathway annotation analyses with permission from Kanehisa Laboratories, Japan (KEGG Copyright Permission reference number 221327) [16, 17]

The Web Gene Ontology Annotation (WEGO) visualization of differentially expressed genes (DEGs) presents genes into three principal GO categories: biological process, cellular component, and molecular function. A total of 37 and 36 terms were enriched in down-regulated and upregulated transcripts respectively (Fig. 2). In both the up- and down- regulated transcripts, cell, cell-part and membrane represented the top three enriched terms under cellular component category. Binding, catalytic activity and molecular transducer activity and transporter activity were the most enriched terms under molecular function category. Under the biological processes category, cellular process, biological regulation, and metabolic process terms were the most enriched in down-regulated transcripts, whereas, cellular process, multicellular organismal process, biological regulation, and metabolic process were the most enriched terms in the up-regulated transcripts (Fig. 2). Pathway analysis of differentially expressed transcripts using the KEGG automated annotation server (KAAS) revealed that ribosome, calcium signalling and neuroactive ligand-receptor interaction were the top three enriched pathways (Fig. 3A, supplementary information S3).

### Transcripts unique to symbiotic and axenic nematodes

**Fig. 4:**
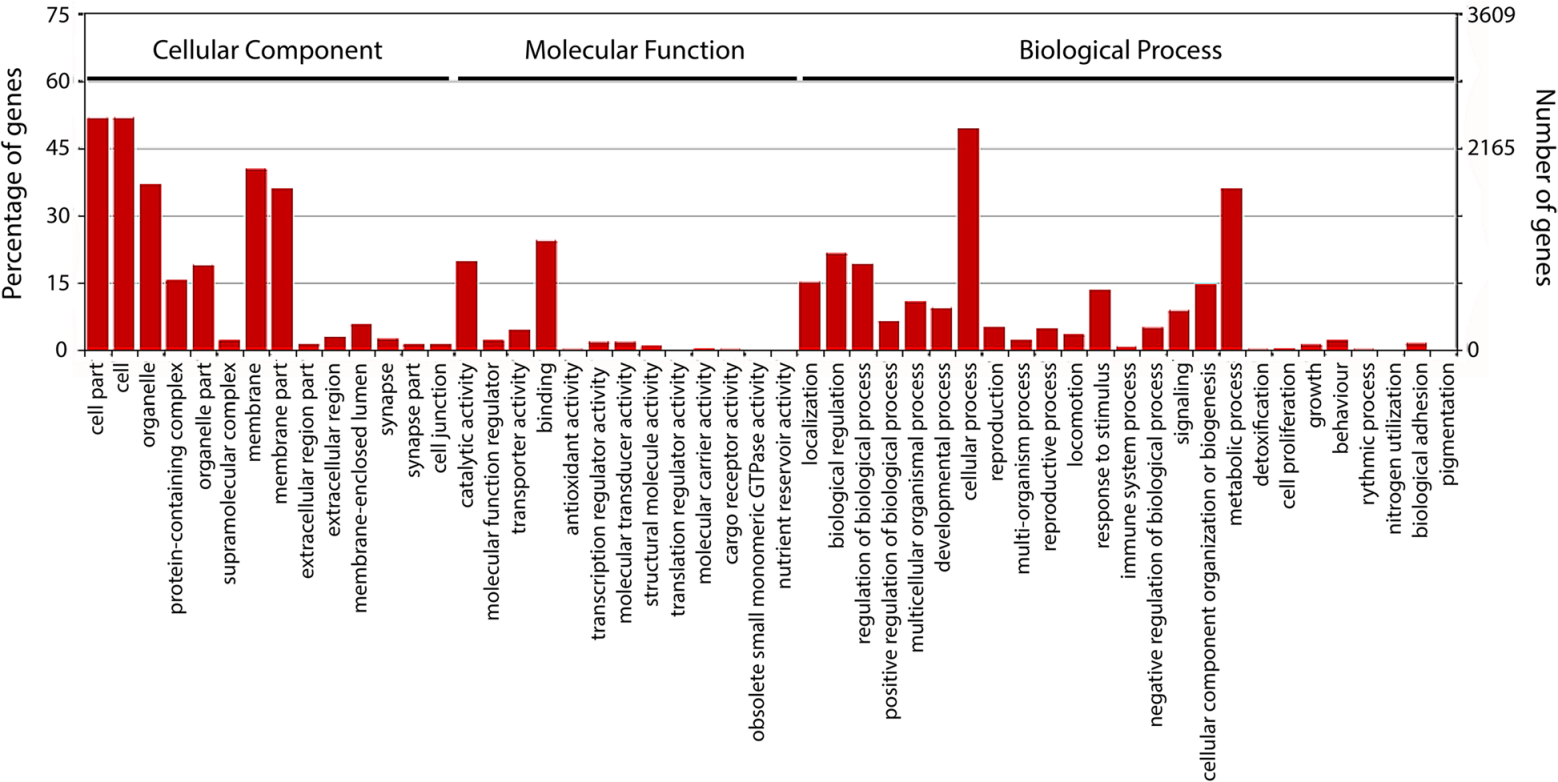
GO annotation of symbiotic-nematode specific transcripts. WEGO (Web Gene Ontology Annotation Plot) tool was used for visualizing, comparing and plotting GO annotation results

As stated above, the results showed that in addition to the 754 differentially expressed transcripts, 12,151 transcripts were unique to symbiotic nematodes, whereas 20 transcripts were unique to axenic nematodes. Annotation of the symbiotic nematode specific transcripts showed enrichment of endocytosis, cAMP signalling pathway, and focal adhesion as the top three enriched pathways (Fig. 3B, supplementary information S3). The WEGO analysis of the symbiotic nematode specific transcripts showed enrichment of 52 terms (Fig. 4). In the cellular component category, cell, cell part and membrane were the top three enriched terms. In the molecular function category, binding, catalytic activity and transporter activity were the top enriched terms, whereas in the biological process category, cellular process, metabolic process and biological regulation were the three most enriched terms (Fig. 4).

**Fig. 5:**
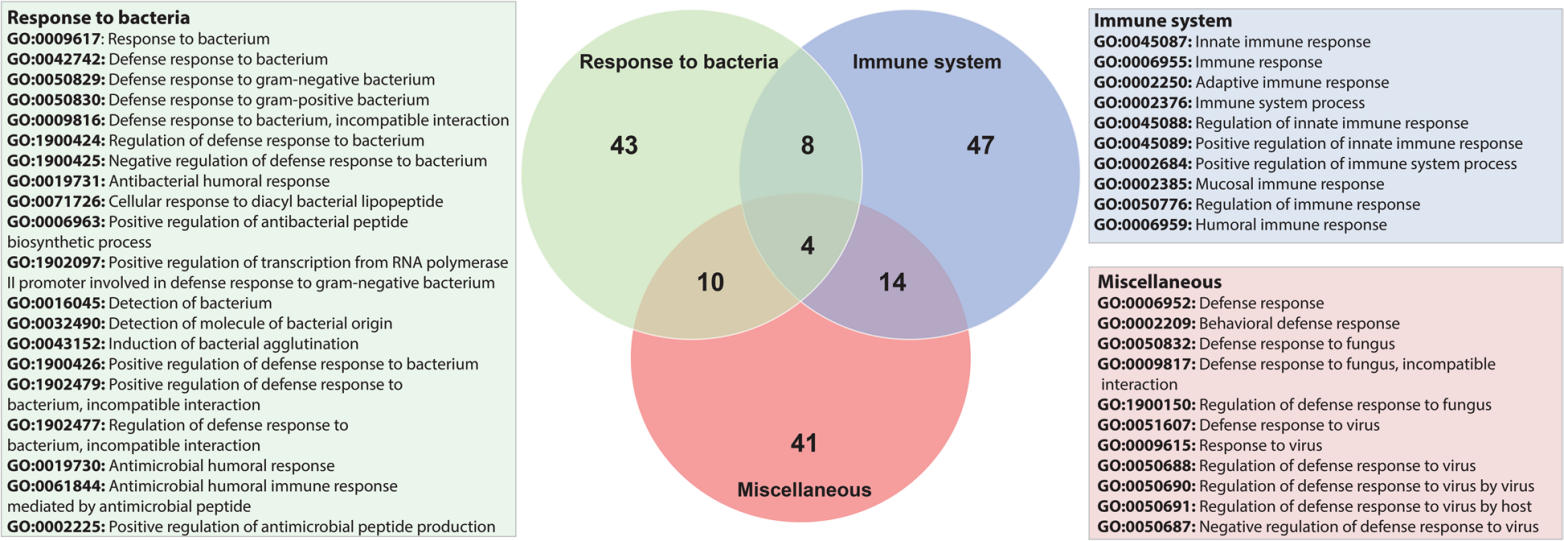
Venn diagram showing specific genes pulled out from symbiotic-nematode specific transcriptome (based on GO annotations) putatively involved in responses to bacteria, immune and defense responses

Since the symbiotic nematodes are interacting with the bacteria, we manually searched for transcripts potentially involved in interaction with bacteria on the basis of annotation, such as GO terms related to responses to bacteria, immune system and defense responses (Fig. 5). A total of 314 transcripts were identified and mapped to 167 genes based on annotations (list of transcripts and gene annotations are provided in supplementary information S4). A total of 65 genes could be grouped into 20 GO terms related to response to bacteria. Seventy-three genes grouped under the terms related to immune system, where the term ‘innate immune response’ (GO:0045087) was the most enriched. All the other terms such as ‘defense response’ (GO:0006952), ‘defense response to fungus’(GO:0050832), ‘defense response to virus’ (GO:0051607) and ‘behavioral defense response’ (GO:0002209) were grouped into ‘miscellaneous category’ containing 69 genes (Fig. 5). Out of 167 genes, 80 genes were assigned to various biological processes highly relevant from the perspective of animal-bacterial interaction (Table 4). Several transcripts belonging to the canonical nematode immune signalling pathways were identified in the symbiotic *H. bacteriophora* transcriptome. From the transforming growth factor β (TGF-β)/DBL-1 pathway- *sma-6*, *sma-3* and *sma-4*; from p38 mitogen-activated protein kinase (MAPK) pathway- *tir-1*, *nsy-1*, *sek-1* and *pmk-1*; from insulin-like receptor (ILR or DAF-2/DAF-16) pathway- *age-1* and *daf-16*; from extracellular signal-regulated kinase (ERK) pathway- *lin-45* and *mek-2* transcripts, and from c-Jun N-terminal kinase (JNK) pathway- *mlk-1, mek-1* and *kgb-1* were detected in the symbiotic nematode’s transcriptome. *abl-1*, *bsk*, and *vhp-1*, which are implicated in stress response pathways were also found in symbiotic nematodes.

**Table 4:** List of genes/proteins important for host-symbiotic bacterial interaction selected based on GO (Gene Ontology) annotations

S. No	Gene/Protein (UniProt identifier)	S. No	Gene/Protein (UniProt identifier)
	**Lipid binding proteins (Immune surveillance and/or effectors)**		**Apoptosis, Autophagy and Endocytosis**
1.	BPI (P17453)	41.	*ced-1* (A8XMW6)
2.	LBP (Q2TBI0)	42.	*ced-3* (P42573)
3.	DMBT1 (Q9UGM3)	43.	*ced-4* (Q60Z52)
4.	CD36 (P26201)	44.	RNF216 (Q9NWF9)
	**Carbohydrate binding proteins (Immune surveillance and/or effectors)**	45.	RAB14 (Q5ZKU5)
5.	*lec-8* (Q09610)	46.	TIAL1 (Q01085)
6.	*irg-7* (A0A131MBU3)	47.	ACD11 (O64587)
7.	CLEC4A (Q9UMR7)	48.	BCL3 (P20749)
8.	LGALS9 (Q3MHZ8)	49.	SKP2 (Q13309)
9.	LY75 (O60449)	50.	*atg-16.2* (Q09406)
10.	CHID1 (Q5EAB4)	51.	*lgg-1* (Q09490)
	**Kinases, Phosphatases and other protein adaptors (Signal transduction)**	52.	*lgg-2* Q23536)
11.	*abl-1* (P03949)	53.	SYT11 (Q9BT88)
12.	*kgb-1* (O44408)	54.	SQSTM1 (Q13501)
13.	*mlk-1* (A0A0K3AV08)	55.	RUBCN (Q92622)
14.	*sek-1* (G5EDF7)	56.	ILRUN (Q5F3N9)
15.	*bsk* (P92208)	57.	C3 (Q2UVX4_
16.	*sma-6* (Q09488)	58.	*faf* (A0A0B4K7S0)
17.	*dkf-2* (O45818)	59.	WASL (O00401)
18.	*dapk-1 (O44997)*	60.	DENND1B (Q6P3S1)
19.	*ksr-1* (G5EFD2)		**Lysozymes**
20.	*pkc-3* (A8WUG4)	61.	*ilys-2* (O76358)
21.	MAPKBP1 (O60336)	62.	*ilys-3* (O76357)
22.	*vhp-1* (Q10038)		**Peroxidases**
23.	*wun* (Q9V576)	63.	*bli-3* (O61213)
24.	PPM1D (O15297)	64.	*hpx-2* (P90820)
25.	*tir-1* (Q86DA5)	65.	*skpo-1* (Q20616)
26.	*sma-3* (P45896)	66.	*gst-5* (Q09596)
27.	*daf-7* (P92172)	67.	*gstk-1* (Q09652)
28.	SMAD3 (P84022)	68.	GLO-3 (Q24JJ8)
	**Transcription factors**		**Transmembrane transporters**
29.	*daf-16* (O16850)	69.	*pgp-1* (P34712)
30.	*elt-2* (Q10655)	70.	*pgp-3* (P34713)
31.	*pnr* (P52168)	71.	SLC17A5 (Q9NRA2)
32.	*fos-1* (G5ECG2)	72.	*aqp-10* (Q09369)
33.	TFEB (P19484)	73.	AQP3 (Q08DE6)
34.	PAX5 (Q02548)		**Mucosa associated protein**
	**Phospholipases (lipolytic)**	74.	MALT1 (Q9UDY8)
35.	PLA2G1B (P00593)	75.	*Cad99C* (Q9VAF5)
36.	PLA2G6 (O60733)	76.	MR1 (C1ITJ8)
37.	*plc-1* (G5EFI8)		**Antimicrobial proteins**
	**Metallopeptidases (proteolytic)**	77.	*acantho1* (Q8I948)
38.	*spg-7* (Q9N3T5)	78.	Antimicrobial protein Ace-AMP1 (Q41258)
39.	*zmp-2* (O44836)	79.	Venom serine protease inhibitor (A0A2R4SV19)
40.	ADAM8 (P78325)	80.	*psidin* (Q17DK2)

Twenty transcripts were found unique to axenic nematodes, out of which nine could be annotated (supplementary information S2). These transcripts coded for putative casein kinase, outer-membrane lipoprotein carrier protein, thermostable monoacylglycerol lipase (MGLP) (bMGL), SH3 domain-containing ysc84-like protein 1, cytochrome c oxidase subunit 3, zinc-specific metallo-regulatory protein, peptide methionine sulfoxide reductase MsrA, probable ccr4-associated factor 1 homolog 1, and unconventional myosin-9b.

### Validation of gene expression patterns by qRT-PCR

**Fig. 6:**
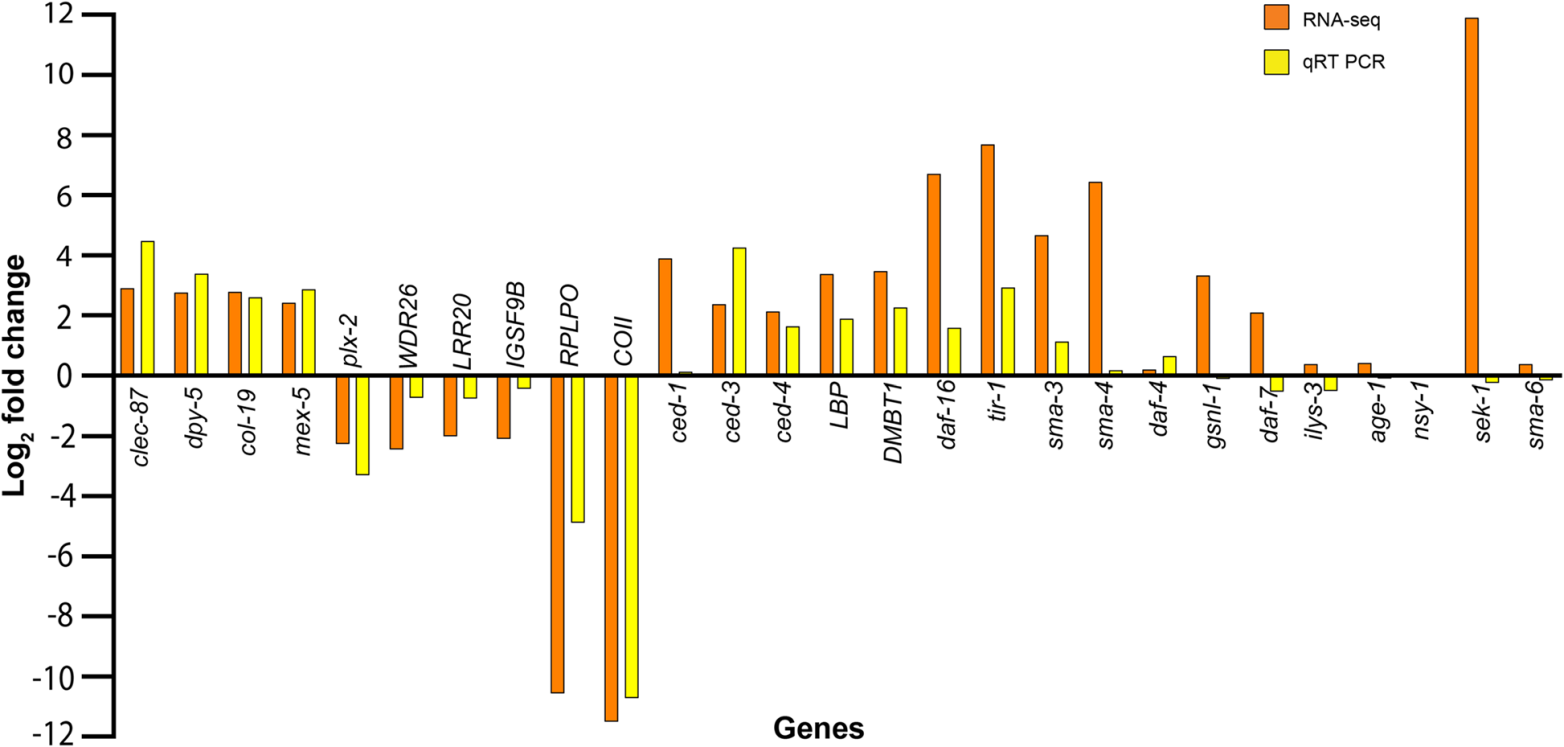
qRT PCR validation of expression patterns of the various differentially expressed and unique transcripts identified in RNA-seq experiment. Most of the tested transcripts conformed to their RNA-seq expression patterns

Expression of 27 randomly selected differentially expressed genes was validated by qRT-PCR. The expression pattern of 20 of the selected genes (*clec-87, dpy-5, col-19, mex-5, plx-2, WDR26, LRR20, RPLP0, COII, ilys-3, age-1, sma-6, ced-3, ced-4, LBP, DMBT1, daf-16, tir-1, daf-4, nsy-1* and) was congruent with the RNA-seq data, although differences were apparent between the expression levels obtained in qRT-PCR and RNA-Seq (Fig. 6). However, seven genes—*ced-1*, *sma-3*, *sma-4*, *daf-7*, *IGSF9B*, *gsnl-1* and *sek-1* which were found significantly up- or down regulated in RNA-seq, were found as expressing at baseline in qRT-PCR analysis (Fig. 6).

## Discussion

*Heterorhabditis* nematodes are widely used for the biological management of insect pests in agriculture. As stated earlier, these entomopathogenic nematodes form a mono-specific symbiotic relationship with *Photorhabdus* bacteria. It has been known that the complex multicellular animals and plants exhibit strong interdependencies with their associated microbes, and these microbes impact animal evolution and various aspects of animal biology [18, 19]. As compared to the animals associated with multiple genera/species of bacteria, the *Heterorhabditis-Photorhabdus* symbiosis offers a powerful model system to investigate symbiosis [20]. Here we used RNA-sequencing to identify *Heterorhabditis* nematode genes and pathways involved in symbiosis with the *Photorhabdus* bacteria. The expression pattern of majority of selected differentially expressed genes could be validated by qRT-PCR. Previous studies have shown that up to 15–20% of genes showing certain expression by RNA-Seq may show ‘non-concordant’ expression when validated by qRT-PCR [21, 22].

### Differentially expressed transcripts

We identified 754 differentially expressed transcripts in the symbiotic nematodes as compared to axenic worms. Analysis of DEGs indicated that the symbiotic bacteria alter the gene expression in the host animals. The ribosomal pathway was the most affected, and the top down-regulated genes were related to ribosomal proteins, confirming a down-regulation of translation in the nematode host. Expression of *COII* encoding ‘Cytochrome c oxidase subunit 2’ and *MT-CYB* encoding ‘Cytochrome b’ were also significantly down-regulated in the symbiotic nematodes. Bacteria are known to affect ribosomal and mitochondrial pathways [23]. As seen in *C. elegans*, nematodes can sense perturbations in the core processes such as blockade of mitochondrial, transcription and translation related pathways, ATP synthesis, proteasomes in intestine or hypodermis, and induce defence responses such as avoidance behaviour and defence genes expression [24–26]. The observed changes in expression levels of core housekeeping genes, for e.g., the downregulation of ribosomal proteins and cytochrome oxidase encoding genes, suggesting slowdown of protein synthesis and cellular respiration, respectively, in our study may be indicative of a trade-off in which elevated immunity is prioritized over other metabolic functions, as seen in other models (for example C*. elegans*-microbe interactions [24–26]).

### Transcripts unique to symbiotic nematodes

Additionally, 12,151 transcripts unique to symbiotic, and 20 unique to axenic nematodes were identified. We ascertained that these transcripts are not contaminants, and filtered the transcripts with FPKM value > 1 prior to further analysis. As opposed to the prevalent approach of analysing only the differentially expressed genes between two conditions (here symbiotic vs. axenic nematodes), we also analysed these 12,151 transcripts and found several transcripts relevant to the phenotype. The symbiotic nematodes are cohabiting with bacterial symbionts, whereas axenic nematodes are not – which represents an extreme difference in the nematode lifestyle. It is possible that some transcripts are exclusively transcribed under symbiotic or axenic conditions. This is in conformation with several research publications on tissue/cell/condition specific transcripts [27–30]. In addition, unique transcripts in symbiotic nematodes might also result from alternative splicing (AS) under specific physiological condition (here presence of bacterial symbionts). Nematodes are known to have AS in the range of 20–30%, typically with 2–3 transcripts per AS locus [31]. However, a deeper analysis of the AS in our experimental conditions is beyond the scope of this study and will be taken up in future.

The transcripts uniquely expressed in axenic nematodes are involved in metabolism (including mRNA deadenylation), transporting lipoproteins and organelles, regulation of signalling pathways, preventing oxidative damage to the cell, and might be important in helping the nematodes adjust to a symbiont-free situation which is not natural for these nematodes. Annotation of the differentially expressed and unique transcripts indicated that several of them are involved in nematode-bacterial interactions through nematode immunity. Host immunity has a vital role in the host-microbe interactions as well as animal growth and development [32–35]. Additionally, the immune system also coordinates biochemical and cellular responses to changes in the molecular landscape in hosts, and helps to maintain a healthy balance between the host and symbionts [36–38].

### Transcripts involved in bacterial recognition

Interactions between hosts and microbes are largely mediated through microbe-derived ligands, i.e., microbe associated molecular patterns (MAMPs) and pattern recognition receptors (PRRs) of hosts [39]. Bacterial factors such as extracellular polysaccharides, lipopolysaccharides, and fimbrial structures are vital to the symbiosis by *Photorhabdus* bacteria [14, 15]. Our results show that the symbiotic nematodes express orthologues of proteins which are known to have pattern recognition receptor activity. For example, orthologs of mammalian proteins such as lipopolysaccharide-binding protein (LBP), bactericidal permeability-increasing protein (BPI), deleted in malignant brain tumors 1 protein (DMBT) / Glycoprotein 340, membrane associated glycoprotein CD36, MHC class I-related protein 1 (MR1) and orthologs of *C. elegans* proteins such as probable galaptin LEC-8, DAF-7 and IRG-7 (an infection response protein) were detected in the transcriptome of symbiotic nematodes. Additionally, several mammalian lectin orthologs with carbohydrate binding activity (for example, CLEC4A (C-type lectin domain family 4 member A), LGALS9 (Galectin-9), and LY75/CLEC13B (Lymphocyte antigen 75) were found expressing in symbiotic nematodes. All these proteins exhibit a range of properties such as lipid binding, carbohydrate binding, and glycolipid binding which play roles in immune surveillance and/or as immunological effectors [40–47].

### Transcripts involved in nematode immunity

Several transcripts expressed in symbiotic nematodes mapped to canonical nematode immune pathways such as MAPK cascades (PMK-1 p38 MAPK, ERK, and JNK pathways), DAF-2/ILR pathway, and TGF-β/DBL-1. Additionally, enrichment of cAMP, mTOR, Rap1, Calcium and Ras signalling pathways was also observed in symbiotic nematodes. A number of recognized kinases, phosphatases, and other proteins (*abl-1, vhp-1, MAPKBP1, PPM1D*) are known to facilitate pathway interactions and/or pathway regulation to achieve immunological balance [48–52]. Furthermore, transcription factors act downstream of immune pathways to regulate gene expression [44, 53]. Transcription factors (TF) such as forkhead TF (*daf-16*), GATA TFs (*elt-2*, *pnr*), *fos-1*, STAT TFs (*sta-1, sta-2*) and TFEB which regulate defense response to bacteria by regulating expression of antibacterial/antimicrobial effectors were also identified in symbiotic nematodes. Transcription factors ELT-2 and TFEB are known to act in tissue-specific manner and regulate defence responses during intestinal infection of nematodes with bacteria [54–56].

### Transcripts encoding antimicrobial mechanisms and effectors

A variety of immune effectors and mechanisms such as autophagy, cell death, endocytosis, production of ROS, lysozymes, proteolytic and lipolytic enzymes and antimicrobial proteins have been implicated in controlling microbial load and activity in host-microbial interactions and play role in defence against infections [44, 53, 57, 58]. In our study, several genes associated with cell death/apoptosis, autophagy and lysozymes (for example: *ced-1*, *ced-3*, *ced-4*, *atg16.2*, *lgg-2*, *ilys-2* and *ilys-3*) were detected exclusively in symbiont-associated nematodes, suggesting these genes could be important in regulating the levels of symbiont population and reduce the deleterious effects of infections on host fitness as seen in other studies [24, 59–63]. Moreover, endocytosis (ko04144) and focal adhesion (ko04510) were the most enriched pathways in symbiotic nematodes. Symbionts that establish a biofilm on the maternal intestine gradually invade the rectal gland cells and become intracellular. Internalization of symbionts has been linked to the endocytosis phenomena [6, 64]. Focal adhesions are integrin containing multi-protein structures. They are associated with the plasma membrane and serve as mechanical linkages between internal actin bundles and the external substrate. Endocytosis along with focal adhesion associated proteins could be exploited by symbionts for attachment and sequestration into host rectal gland cells. This kind of molecular interaction has been studied in *Staphylococcus aureus*, which uses the integrin pathway and focal adhesion kinases to attach to host cells and for internalization [65, 66]. Several peroxidase encoding genes such as *bli-3*, *hpx-2*, *skpo-1*, *gst-5*, *gstk-1* and *glo-3* were detected in symbiotic nematodes. Peroxidases catalyse the generation of ROS (reactive oxygen species), which are well known microbicidal effectors and signalling molecules [67–69]. Numerous studies in invertebrates have implicated the role of ROS in regulation of gut microbiota [57, 70]. *Photorhabdus* symbionts are known to utilize pentose phosphate pathway to avoid oxidative stress and hence persist in their animal hosts [71] which is supported by the expression of many peroxidases and reductases in symbiotic nematodes.

In the context of symbiont evolution, animal-microbe symbiotic systems have been classified as open, closed and mixed [72]. In the open systems (e.g., bobtail squids and *Aliivibrio fischeri*), microbes colonise their hosts repeatedly from external environmental niches and both the partners manipulate each other at molecular level to achieve symbiosis. The closed systems (e.g., aphids and *Buchnera aphidocola*) are characterised strictly by maternal transmission of symbionts enforcing clonality and a reduction of host immune responses against symbionts [35]. In the mixed systems (e.g., common fruit fly and *Wolbachia pipientis*), vertical transmission is the rule, with occasional horizontal transmission of symbionts [72]. Based on the nematode-bacteria symbiotic life cycle [6, 73], species associations and evolutionary history [74, 75], and a large number of molecular perturbations observed in our results—it may be suggested that the *Heterorhabditis* nematode-*Photorhabdus* bacteria symbiosis is a mixed symbiotic system, and exhibits characteristics of an evolving symbiosis.

## Conclusions

**Fig. 7:**
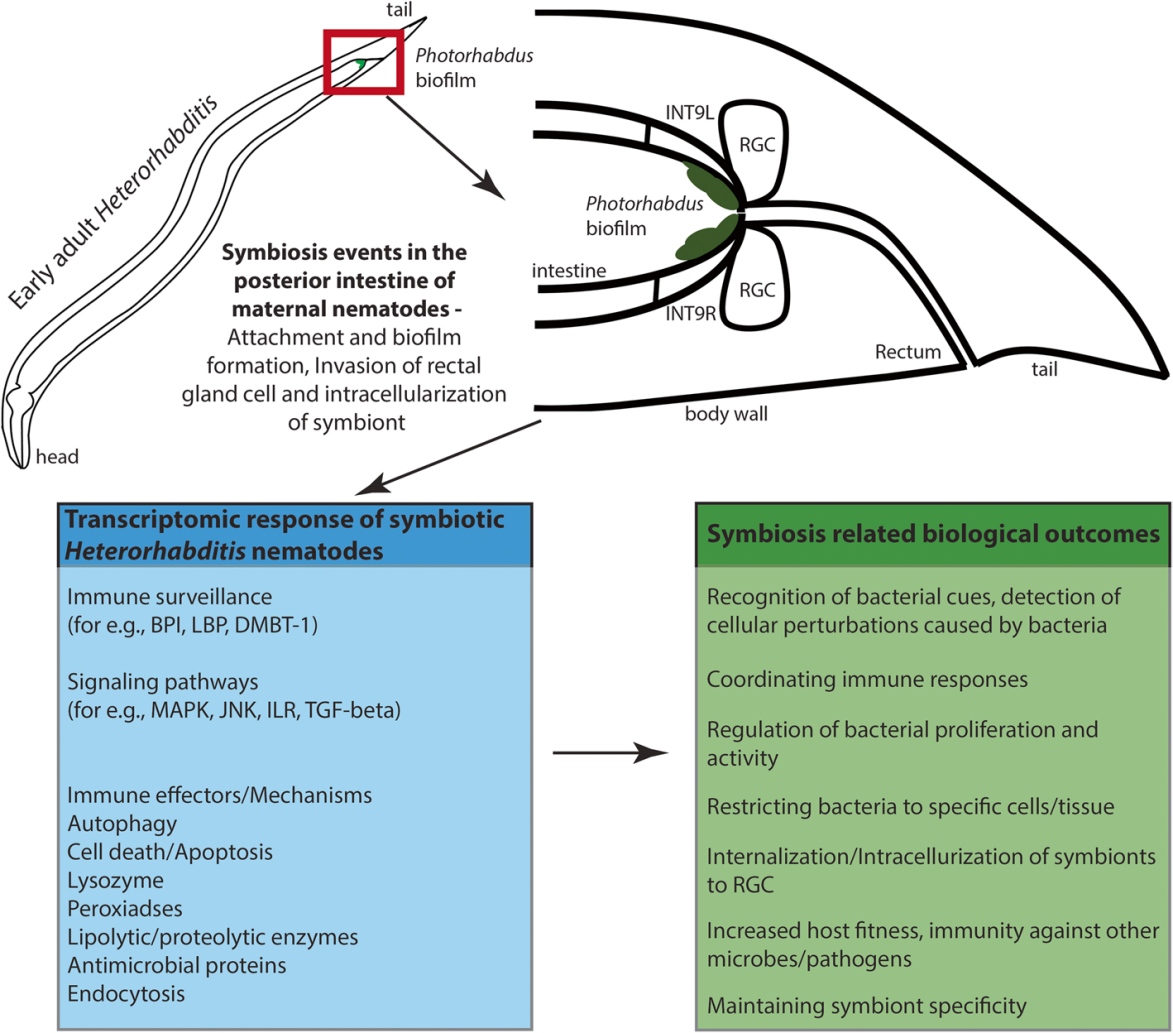
A model presenting symbiotic events and the transcriptomic responses in early-adult stage of maternal nematodes, and their interpretation in relevance to symbiosis with *Photorhabdus* bacteria. Colonization processes involve two-way crosstalk between host and symbiont. From the nematode host side, based on gene expression profiling, expression of immune system components which are involved in the recognition of bacteria, bacteria-derived molecules and detection of any cellular perturbations caused by bacteria are observed. It leads to activation of signalling pathways and results in an array of host immune and defence responses such as autophagy, apoptosis and production of antimicrobial proteins. These effectors and mechanisms regulate symbiont bacterial numbers and help achieve successful symbiosis. INT9L/INT9R – posterior nematode intestinal cells; RGC- rectal gland cells

The transcriptomes of symbiotic early-stage *Heterorhabditis* nematodes were compared to axenic nematodes. A total of 754 differentially expressed transcripts were found in symbiotic nematodes as compared to axenic nematodes, whereas 12,151 transcripts unique to symbiotic nematodes were also observed. The symbiotic *Heterorhabditis* nematodes respond to presence of symbiotic bacteria by expressing various transcripts involved in a multi-layered immune response which includes sensing the bacteria, activation of canonical immune pathways and production of antimicrobials. These responses observed in symbiotic nematodes might represent non-systemic and evolved localized responses to maintain mutualistic bacteria at non-threatening levels. A model indicating the nematode factors involved in achieving and maintaining bacterial symbiosis is proposed (Fig. 7). Our findings suggest that *Heterorhabditis* nematode immune system plays a pivotal role in maintenance of symbiosis with its *Photorhabdus* bacterial partner.

## Materials and methods

### Nematode and bacterial strains and culture media

The nematode *H. bacteriophora* strain TTO1 used in this study was a gift from Dr. Byron J. Adams (Brigham Young University, USA) to (Late) Dr. (Mrs.) Sudershan Ganguly. Nematode stocks were maintained in the laboratory by infecting the last instar larvae of the greater wax moth, *Galleria mellonella* with infective juveniles (IJs) using standard procedures [76]. Freshly emerged IJs were collected in Ringer’s solution (100 mM NaCl, 1.8 mM KCl, 2 mM CaCl_2_, 1 mM MgCl_2_ and 5 mM HEPES, pH 7.0) using modified White’s trap, stored at 15 °C and used within 10 days. Bacterial strains used in this study were *P. luminescens* ssp. *laumondii* strain TTO1 (wild type symbiont of *H. bacteriophora* strain TTO1), *P. luminescens* ssp. *laumondii* strain TTO1GFP (TTO1 containing Tn7-GFP [6]), and *P. luminescens* ssp*. laumondii* strain TTO1 *ΔmadA* (GFP labelled transmission defective mutant that is unable to colonize the nematodes [15]). Modified Luria broth (LB) medium (in one litre—casein enzymic hydrolysate 10 g, yeast extract 5 g, sodium chloride 10 g, sodium pyruvate 1 g) was used for preparation of starter cultures of *Photorhabdus*. The nematodes were grown on lawns of *Photorhabdus* on nematode growth media (NGM) (in one litre—peptone 5 g, sodium chloride 5 g, meat extract B 1.50 g, yeast extract 1.50 g, agar 16 g) supplemented with 1 ml of cholesterol solution (5 mg/ml).

Symbiotic *H. bacteriophora* strain TTO1 IJs were cultured on lawns of wild type *P. luminescens* ssp. *laumondii* strain TTO1. For the preparation of nematode eggs, ~ 400 surface sterilized IJs were added to bacterial lawns and incubated at 28 °C for 3–4 days. Gravid adult hermaphrodites were washed off the lawns with sterile distilled water, and added to axenizing solution (2.4% (v/v) NaOCl, 0.25 N KOH). The eggs were washed and collected as described earlier [77]. *Heterorhabditis*-*Photorhabdus* symbiosis is obligate in nature, however, it is possible to culture both the partner separately under the laboratory condition. These nematodes cannot be multiplied/cultured on any other bacteria such as *E. coli*. One of the reported methods to obtain axenic *Heterorhabditis* is to grow them on transmission defective symbiont bacterial strain (for example [15, 78]). Here isolated nematode eggs were placed on the lawns of the transmission mutant *Photorhabdus* strain *ΔmadA* to generate symbiont free axenic IJs [15]*.* To confirm the sterility of axenic nematodes, the IJs were surface sterilized in 1% commercial bleach for 5 min, homogenized, and plated on Luria Bertani agar (in one litre—casein enzymic hydrolysate 10 g, yeast extract 5 g, sodium chloride 10 g, agar 10 g). Antibiotics were added to maintain axenic nematodes stock at the following concentrations- streptomycin 100 mg/ml, ampicillin 100 mg/ml, kanamycin 30 mg/ml and gentamicin 10 mg/ml.

### Preparation of nematodes for RNA-Sequencing experiment

Symbiont bacterial attachment and biofilm formation takes place in the posterior nematode intestinal cells when the nematode is at early-adult stage, i.e., 36–40 h after IJ comes out of diapause [6]. The symbiotic (S) and axenic (A) IJs were obtained by growing nematodes on the lawns of GFP-labelled *P. luminescens* ssp. *laumondii* and symbiosis-defective *Photorhabdus* mutant *ΔmadA* strain for 36–40 h, respectively. *ΔmadA* supports the growth of nematode but do not colonize the nematode gut and is not transmitted to the progeny, therefore the nematodes remain free of symbionts [15]. Further, the nematodes were starved for 4 h to eliminate transient cells in nematode intestine prior to imaging. The presence or absence of symbiotic bacterial biofilm (cells of persistent green colonizing bacteria) in the nematodes was determined by fluorescent microscopy using a ZEISS microscope (ZEISS Axio Imager.Z2 ACR Research Upright Microscope from Carl Zeiss, Germany). The nematodes were washed from the plates, filtered using 20-micron sieves to get rid of bacteria attached to the nematode surface, collected in trizol and snap-frozen for RNA isolation. The experiments were repeated thrice.

### RNA isolation, cDNA synthesis, library preparation and RNA-sequencing

Total RNA was extracted from a 100 μl pellet of symbiotic and axenic early-adults of *H. bacteriophora* separately by TRIzol Plus RNA Purification Kit (Thermo Fisher Scientific, Massachusetts, USA) according to the manufacturer’s instructions. RNA was treated with DNase I, amplification grade (Thermo Fisher Scientific, Massachusetts, USA) to remove any genomic DNA contamination. Integrity of the extracted RNA was tested on an Agilent 2100 Bioanalyzer (Agilent Technologies, Santa Clara, CA, USA) and RNA with an RNA integrity number (RIN) of 8.0 or above was used for messenger RNA (mRNA) purification. The mRNA was purified from approximately 5 μg of intact total RNA using oligodT beads (Illumina® TruSeq® RNA Sample Preparation Kit v2). The purified mRNA was fragmented in the presence of bivalent cations and first strand cDNA was synthesized using Superscript II reverse transcriptase (Invitrogen, Carlsbad, CA, USA) and random hexamer primers (Invitrogen, Carlsbad, CA, USA). Second strand cDNA was synthesized by DNA polymerase I and RNaseH following standard protocol (Illumina, TrueSeq). The cDNA was cleaned using Agencourt AMPure XP purification kit (Beckman-Coulter, Brea, CA, USA), amplified, quantified using a Nanodrop spectrophotometer and checked for quality with a Bioanalyzer. In total, 6 libraries were prepared for symbiotic and axenic nematode samples (3 each) as per the Illumina protocols. cDNA libraries were sequenced on Illumina HiSeq platform (150 × 2 Paired-End format) by outsourcing to Bionivid Technologies Pvt. Ltd., Bengaluru, India.

### Bioinformatic analyses

*H. bacteriophora* genome available on Wormbase Parasite (BioProject ID: PRJNA13977, [78]) is fragmented (BUSCO score for assembly-Complete 87.1%, Fragmented 9.6%). In addition, gene prediction and annotation of this assembly is poor (BUSCO score for annotation – Complete-31.4%, Fragmented 14.9%). One other genome assembly published for *H. bacteriophora* strain G2a1223 [79] is not available in the public domain. Under these circumstances, we generated a *de-novo* transcriptome assembly for the present study. The bioinformatic analysis methods pipeline is provided in supplementary figure S3. All the Paired-end fastq reads were subjected to adapter trimming and quality filtering using fastp (v.0.20.0) [80] at minimum length cutoff of 70 bp and phred quality score of 30. High quality (HQ) reads from all the samples were pooled together to generate a *de-novo* primary assembly using Trinity Assembler (v.2.4.0) [81]. Reads were *in-silico* normalized and the maximum read coverage was set at 80. A minimum count of Kmer for Inchworm assembly was set at 7 (parameters: –normalize_max_read_cov = 80 and –min_kmer_cov = 7). Nucleotide sequences (transcripts) which were similar in length and identity were clustered together using CD_HIT_EST tool with sequence identity threshold of 80% (-c = 0.8) and length difference cutoff of 80% (-s = 0.8) [82], and only one representative sequence (unigene) per cluster was retained. The assembly was curated further using Bionivid’s in-house amelioration pipeline Transimprove [83] to get high quality transcripts supported by adequate depth (5x) and coverage (70%). To make sure that no contaminating sequences are used for downstream analysis, the sequences were screened using NCBI taxonomy server. Additionally, FastQC v0.11.9 screen against *Photorhabdus* genome was done to check for possible symbiont bacterial contamination [7, 84]. Assembly validation was done by mapping the reads against the *de-novo* transcriptome using Kallisto v0.46.1 [85]. The completeness of transcriptome assembly was assessed by BUSCO v5.4.2 against eukaryota_odb10.2019–11-20 and nematoda_odb10.2019–11-20 databases [86]**.**

The transcripts were quantified with RSEM v1.1.17 method [87] and the expression was normalized using fragment per kilobase million (FPKM) metric [88]. The expression data from each of the replicates were analysed using the GGally v1.5.0 R package which utilizes Pearson correlation to determine correlation between the replicates. Differential gene expression analysis was performed using DESeq package v1.30.1 in R language [89]. DESeq provides method to test differential expression by use of the negative binomial distribution. Read counts taken from alignment bam files were taken as input, and differential analysis was performed by comparing transcriptome of symbiotic nematodes to axenic nematodes. Transcripts with log_2_ fold change ≥ 2 and *p-value* ≤ 0.05 were considered as significantly up-regulated, while those with log_2_ fold change ≤ -2 and *p-value* ≤ 0.05 were treated as significantly down-regulated. Hereafter, the up-regulated or down-regulated transcripts refer to transcripts up- /down-regulated in symbiotic nematodes (treatment) as compared to the axenic nematodes (control). Sample-specific transcripts were identified based on the normalized expressions. A FPKM > 1 cut-off was used to filter the false positives and identify the uniquely expressed transcripts in the symbiotic and axenic nematodes. The transcripts were annotated by blastx search against protein sequences from UniProt, SwissProt and NCBI RefSeq databases. Further, Trinotate v3.0.0 (Trinotate/Trinotate.github.io), a tool for functional annotation of transcriptome was also used to annotate the un-annotated transcripts. Filtration criteria used for blastx were: E-value ≤ 0.001, query coverage ≥ 60 and percentage identity ≥ 40. BLAST results were further processed using Blast2GO (v1.3) and KEGG Pathway analysis [16] was done on KAAS (KEGG Automatic Annotation Server) annotation server (https://www.genome.jp/kegg/kaas/) [17, 90]. KAAS provides functional annotation of genes by BLAST comparisons against the manually curated KEGG GENES database (https://www.kegg.jp/kegg/). The result contains KO (KEGG Orthology) assignments and automatically generated KEGG pathways. We used the BBH (bi-directional best hit) method to assign orthologs. The Database for Annotation, Visualization and Integrated Discovery (DAVID) bioinformatics resource (v.6.7) was used for gene ontology based functional annotation and pathway enrichment analysis for which RefSeq IDs of sequences were used as input [91]. WEGO (https://biodb.swu.edu.cn/cgi-bin/wego/index.pl), a web-based tool for visualization of gene ontology [92] was used to plot the gene ontology results obtained using DAVID.

### Validation of gene expression patterns by qRT-PCR

The expression pattern of 27 randomly selected transcripts was validated by quantitative real-time PCR (qRT-PCR). cDNA was prepared by reverse transcription of 500 ng of the RNA by Superscript VILO (Invitrogen, Carlsbad, CA, USA). qRT-PCR was performed on a Realplex2 thermal cycler equipment (Eppendorf, Hamburg, Germany) using SYBR Green Supermix Kit (Eurogentec, Liege, Belgium). A constitutively expressed nematode gene, 18S rRNA, was used as an internal reference. Three biological and three technical replicates were maintained for each sample. Data were analyzed by the ΔΔCt method [93], and the results were expressed as log_2_-transformed fold change values. The oligonucleotide primers used for qRT-PCR are listed in supplementary table S2.

## Supplementary Information

**Additional file 1:** **Supplementary table S1.** Correlation coefficient between various replicates of the RNA-seq samples. **Supplementary table S2.** List of primers used in the qRT-PCR validation. **Supplementary figure S1.** Donut chart representing the Gene Ontology (GO) and KAAS (KEGG Automatic Annotation Server) functional annotations of all the transcripts pooled from the treatment (symbiotic nematodes) and control (axenic nematodes) groups. The top 10 enriched gene ontologies (GO) terms under each category of cellular component, biological process and molecular function (the inner circle) and top 10 ten enriched KEGG pathways are represented. The relative area under each GO term/KEGG ID indicates the number of transcripts mapping to that GO term/KEGG ID. **Supplementary figure S2.** **A**. Heat map showing the relative expression of transcripts in symbiotic and axenic early-adults of *Heterorhabditis*. The map was generated by using the FPKM values, and all the three replicates (R1-R3) are presented. **B.** Volcano plots representing differentially expressed transcripts (log_2_ fold change) in symbiotic nematodes as compared to axenic nematodes. Out of 754 differentially expressed transcripts, 547 transcripts were down-regulated, whereas, 207 transcripts were up-regulated in symbiotic nematodes. **Supplementary figure S3.** Bioinformatic analysis pipeline used to analyse the RNA-Seq data.

**Additional file 2:** **Supplementary information 2A.** A list of all the *H. bacteriophora* transcripts identified in the experiment. **Supplementary information 2B.** A list of the differentially expressed transcripts in symbiotic *H. bacteriophora* nematodes as compared to axenic *H. bacteriophora* nematodes. **Supplementary information 2C.** The transcripts unique to symbiotic *H. bacteriophora* nematodes. **Supplementary information 2D**. The transcripts unique to axenic *H. bacteriophora* nematodes.

**Additional file 3:** **Supplementary information S3A.** KAAS and KEGG based pathway enrichment analysis for all the transcripts in *H. bacteriophora*. **Supplementary information S3 B.** KAAS and KEGG based pathway enrichment analysis for the differentially expressed transcripts in symbiotic *H. bacteriophora* nematodes as compared to axenic nematodes. **Supplementary information S3 C.** KAAS and KEGG based pathway enrichment analysis for the transcripts unique to symbiotic *H. bacteriophora* nematodes.

**Additional file 4:** **Supplementary information S4.** Putative symbiosis relevant genes of *H. bacteriophora* identified on the basis of annotations.

## Data Availability

Raw sequence data has been deposited in Sequence Read Archive, with accession numbers SRR14162364, SRR14162365, SRR14162366, SRR14162367, SRR14162368, and SRR14162369. Bio-sample accession number is SAMN18646821, and Bio-project accession number is PRJNA720314. This Transcriptome Assembly has been deposited at DDBJ/EMBL/GenBank under the accession GKBO00000000. The version described in this paper is the first version, GKBO01000000. All the data associated with this manuscript can be accessed through the Bioproject link https://www.ncbi.nlm.nih.gov/bioproject/?term=PRJNA720314.

## Abbreviations

RNA-Seq: RNA sequencing
HQ reads: High quality reads
cDNA: Complementary DNA
GO: Gene ontology
WEGO: Web gene ontology annotation plot
DEGs: Differentially expressed genes
IJ: Infective juvenile
KAAS: KEGG automated annotation server
KEGG: Kyoto encyclopedia of genes and genomes
qRT PCR: Quantitative real time PCR
EPN: Entomopathogenic nematodes
