# Supplementary material for: RNA-Sequencing of Heterorhabditis nematodes to identify factors involved in symbiosis with Photorhabdus bacteria
Source: BMC Genomics. 2022 Nov 7;23:741. doi: 10.1186/s12864-022-08952-4 (PMC9639317; doi:10.1186/s12864-022-08952-4)

**Supplementary information 1. Supplementary tables S1-S2, supplementary figure S1-S3.**

**Supplementary table S1:** Correlation coefficient between various replicates of the RNA-seq samples

| **Axenic nematode Sample** | | | | **Symbiotic nematode Sample** | | | |
| --- | --- | --- | --- | --- | --- | --- | --- |
| Sample | Axenic (Rep 1) | Axenic (Rep 2) | Axenic (Rep 3) | Sample | Symbiotic (Rep 1) | Symbiotic (Rep 2) | Symbiotic (Rep 3) |
| Axenic (Rep 1) | 1 | 0.95 | 0.83 | Symbiotic (Rep 1) | 1 | 0.77 | 0.92 |
| Axenic (Rep 2) | 0.95 | 1 | 0.89 | Symbiotic (Rep 2) | 0.77 | 1 | 0.87 |
| Axenic (Rep 3) | 0.83 | 0.89 | 1 | Symbiotic (Rep 3) | 0.92 | 0.87 | 1 |

**Supplementary table S2:** List of primers used in the qRT-PCR validation

| **S. No.** | **Gene annotation** | **Primer sequence (5’-3’)** | **Product length (bp)** | **Tm**  **°C** | **Transcript ID** |
| --- | --- | --- | --- | --- | --- |
| 1 | *clec-87* | FP- CGACTGAGGAGCCAACTACC | 181 bp | 60 | TRINITY_DN1416_c0_g2_i1--LEN=1071 |
|  |  | RP- TTCGCATAAACCGATGATGA |  |  |  |
| 2 | *dpy-5* | FP- ACAAATGGCCGTTGATAAGG | 153 bp | 60 | TRINITY_DN5772_c0_g1_i1--LEN=1068 |
|  |  | RP- CCTGTCCGGCTATCATTCAT |  |  |  |
| 3 | *col-19* | FP- GCTTTGTGTTTCGTTGACCA | 180 bp | 60 | TRINITY_DN7746_c0_g4_i1--LEN=1358  TRINITY_DN821_c0_g1_i1--LEN=940 |
|  |  | RP- GGATTTCCAGGCCTACCTTC |  |  |  |
| 4 | *mex-5* | FP- AGAAAGAAAACGGACCACGA | 156 bp | 60 | TRINITY_DN5388_c0_g2_i2--LEN=2237  TRINITY_DN5388_c0_g2_i3--LEN=2310  TRINITY_DN5388_c0_g2_i6--LEN=2088  TRINITY_DN5388_c0_g2_i8--LEN=2258 |
|  |  | RP- CACGCAGATCTTCAGTTCCA |  |  |  |
| 5 | *plx-2* | FP- CGTGGGAGACTCTGGTCAAT | 203 bp | 60 | TRINITY_DN4092_c0_g1_i1--LEN=4657 |
|  |  | RP- ATTTTTGCACTGTCCCCTTG |  |  |  |
| 6 | *WDR26* | FP- GGTCTGTAAACCACCGCCTA | 240 bp | 60 | TRINITY_DN7542_c0_g1_i2--LEN=1259 |
|  |  | RP- TTGGCAGCATTCAGTTCTTG |  |  |  |
| 7 | *LRR20* | FP- CACTTTCCCTTGTTGGATGG | 225 bp | 60 | TRINITY_DN6804_c0_g1_i6--LEN=3224 |
|  |  | RP- GGTTAACAGGAGGCACCGTA |  |  |  |
| 8 | *IGSF9B* | FP- AGCGATGGACCATTCAGTTC | 236 bp | 60 | TRINITY_DN6322_c0_g2_i1--LEN=2942 |
|  |  | RP- TTACGATGGGAGCCAGTACC |  |  |  |
| 9 | *RPLP0* | FP- GGGAAGACAGATCTACCTGGAA | 168 bp | 60 | TRINITY_DN3587_c0_g1_i1--LEN=1046 |
|  |  | RP- CCCCCTCCAAGTTCAAATC |  |  |  |
| 10 | *COII* | FP- AGGTTACAGGGCATCAGTGG | 197 bp | 60 | TRINITY_DN6295_c0_g10_i1--LEN=742 |
|  |  | RP- TCAAGCATGAATAACATCAGCA |  |  |  |
| 11 | *ced-1* | FP- TCCAACAGCTGTCCTGATTG | 169 bp | 60 | TRINITY_DN7259_c0_g1_i27--LEN=2471  TRINITY_DN7259_c0_g1_i17--LEN=3809 |
|  |  | RP- CCCAGTAGCCCGGTATACAA |  |  |  |
| 12 | *ced-3* | FP- CAAGGCACAGAAGTCGATCA | 175 bp | 60 | TRINITY_DN5119_c0_g4_i1--LEN=522  TRINITY_DN5119_c0_g3_i1--LEN=872 |
|  |  | RP- CATGGGTAAGAACCACGACA |  |  |  |
| 13 | *ced-4* | FP- GATCTGCCACATTTCAAGCA | 170 bp | 60 | TRINITY_DN7359_c0_g1_i10--LEN=717  TRINITY_DN7359_c0_g1_i9--LEN=1738  TRINITY_DN7359_c0_g1_i14--LEN=873  TRINITY_DN7359_c0_g1_i13--LEN=1985 |
|  |  | RP- GCATCACGCGTAGTAGCAAG |  |  |  |
| 14 | *LBP* | FP- GGATCAGCAACACGCAGATA | 189 bp | 60 | TRINITY_DN5483_c0_g1_i3--LEN=1784 |
|  |  | RP- TCCCTTGCATAGTTGGCATA |  |  |  |
| 15 | *DMBT1* | FP- CACACCCATATGCCAGTGAT | 172 bp | 60 | TRINITY_DN7168_c2_g5_i1--LEN=832 |
|  |  | RP- TGTGATCCCGATAATTTTGC |  |  |  |
| 16 | *daf-16* | FP- TCCTGATGGCAACAACACAT | 159 bp |  | TRINITY_DN7792_c0_g1_i15--LEN=2589  TRINITY_DN7792_c0_g1_i2--LEN=2913 |
|  |  | RP- TCGGCGTAGCTCATATTTCC |  |  |  |
| 17 | *tir-1* | FP- CTCCATTGGCTGGATACGAT | 148 bp |  | TRINITY_DN7733_c0_g4_i1--LEN=3597 |
|  |  | RP- TTTCCATCATCTGTCGGTGA |  |  |  |
| 18 | *sma-3* | FP- AAACCTCTCGGAGTCGTCAA | 148 bp | 60 | TRINITY_DN6489_c0_g1_i4--LEN=1758  TRINITY_DN6489_c0_g1_i5--LEN=1059  TRINITY_DN6489_c0_g1_i2--LEN=1842 |
|  |  | RP- GGCACGTTGTAGCATGTGAT |  |  |  |
| 19 | *sma-4* | FP- TGCCTAATGGTTGTGTCGAG | 128 bp | 60 | TRINITY_DN8082_c0_g7_i2--LEN=1719  TRINITY_DN7882_c0_g1_i19--LEN=831  TRINITY_DN7882_c0_g1_i5--LEN=545  TRINITY_DN8082_c0_g7_i3--LEN=1771  TRINITY_DN8082_c0_g7_i4--LEN=1068 |
|  |  | RP- GCCATCCTTGGCTGTTAGAC |  |  |  |
| 20 | *daf-4* | FP- AAGAGGAAAGGCTGGAGAGG | 130 bp | 60 | TRINITY_DN6869_c0_g1_i2--LEN=2335 |
|  |  | RP- AGCAGCCAATCCAAAATCAG |  |  |  |
| 21 | *gsnl-1* | FP- CGGCGGATATGATACTGGTT | 168 bp | 60 | TRINITY_DN4551_c0_g2_i3--LEN=723 |
|  |  | RP- ATATCGCGACCCAAATCAAG |  |  |  |
| 22 | *daf-7* | FP- TTTATCGTCGCACCACTCAG | 152 bp | 60 | TRINITY_DN5392_c0_g2_i5--LEN=597 |
|  |  | RP- CAGCATTGCATAAGGTGCTC |  |  |  |
| 23 | *ilys-3* | FP- TCTATCGCTACCGCTGACAA | 172 bp | 60 | TRINITY_DN6977_c0_g2_i1--LEN=2116  TRINITY_DN6977_c0_g2_i9--LEN=1285 |
|  |  | RP- TAATATCCTCGCCCGATTTG |  |  |  |
| 24 | *age-1* | FP- ACATGGGTGCCTCAGATCAT | 120 bp | 60 | TRINITY_DN7607_c0_g1_i7--LEN=613  TRINITY_DN7607_c0_g1_i6--LEN=1756 |
|  |  | RP- CTAGCCACCTGGGATTGTGT |  |  |  |
| 25 | *nsy-1* | FP- AAGCGGTTAGCTGGATTGAA | 159 bp | 60 | TRINITY_DN7835_c1_g1_i2--LEN=5349  TRINITY_DN7835_c1_g1_i3--LEN=3888  TRINITY_DN7835_c1_g1_i5--LEN=1543  TRINITY_DN7835_c1_g1_i8--LEN=5287 |
|  |  | RP- AGGTCTTCCCGTAGCCATTT |  |  |  |
| 26 | *sek-1* | FP- TGGGCTAACAGCGATTATGA | 141 bp | 60 | TRINITY_DN6553_c0_g1_i10--LEN=1921 |
|  |  | RP- TCGAGGACTAGGCTCCTTCA |  |  |  |
| 27 | *sma-6* | FP- AGCCAAATACAGAGGCAGCTA | 143 bp | 60 | TRINITY_DN8152_c1_g1_i2--LEN=1919  TRINITY_DN8152_c1_g1_i4--LEN=1147  TRINITY_DN8152_c1_g1_i5--LEN=2620 |
|  |  | RP- TGCTAATATCAGCCGCAACA |  |  |  |

**Supplementary figure S1:** Donut chart representing the Gene Ontology (GO) and KAAS (KEGG Automatic Annotation Server) functional annotations of all the transcripts pooled from the treatment (symbiotic nematodes) and control (axenic nematodes) groups. The top 10 enriched gene ontologies (GO) terms under each category of cellular component, biological process and molecular function (the inner circle) and top 10 ten enriched KEGG pathways are represented. The relative area under each GO term/KEGG ID indicates the number of transcripts mapping to that GO term/KEGG ID.


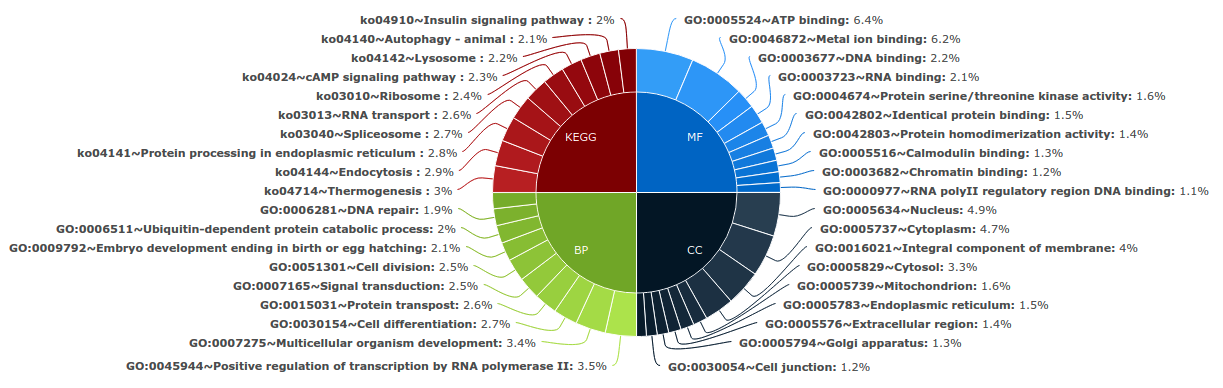


**Supplementary figure S2: A**. Heat map showing the relative expression of transcripts in symbiotic and axenic early adults of *Heterorhabditis*. The map was generated by using the FPKM values, and all the three replicates (R1-R3) are presented. **B.** Volcano plots representing differentially expressed transcripts (log2 fold change) in symbiotic nematodes as compared to axenic nematodes. Out of 754 differentially expressed transcripts, 547 transcripts were down-regulated, whereas, 207 transcripts were up-regulated in symbiotic nematodes.


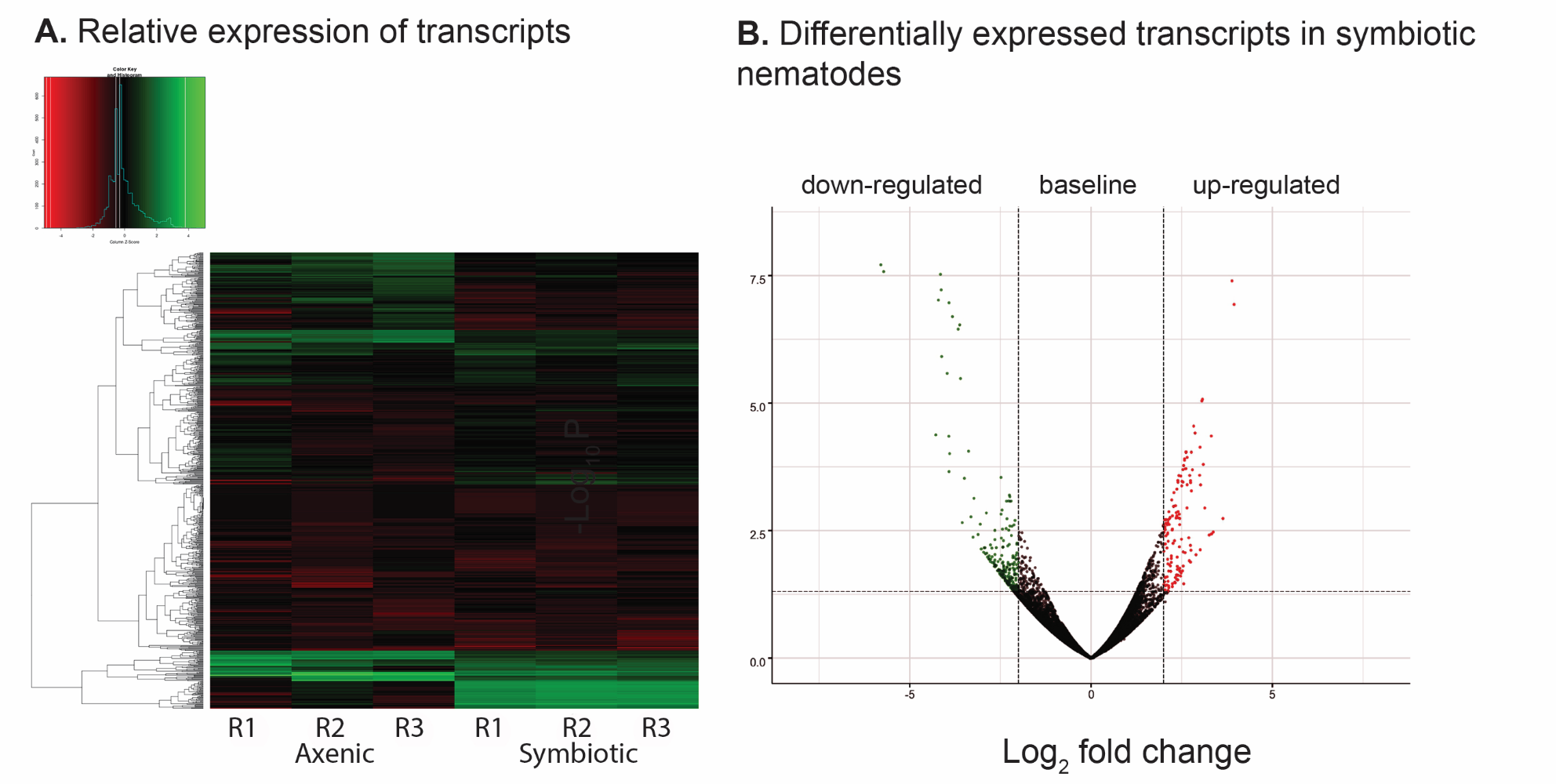


**Supplementary figure S3:** Bioinformatic analysis pipeline used to analyse the RNA-Seq data.


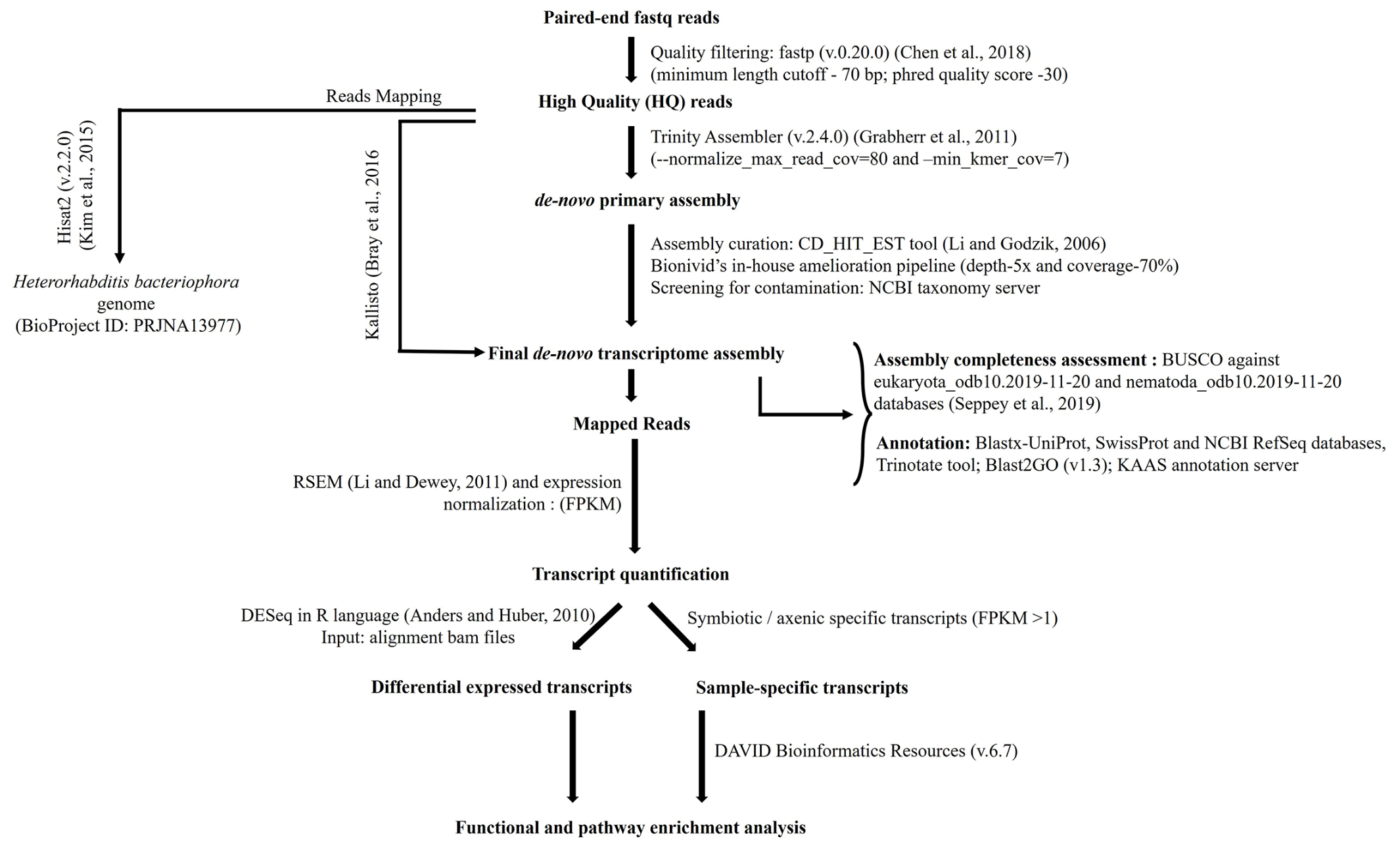

Supplement: Supplementary file 1 — Additional file 1: Supplementary table S1. Correlation coefficient between various replicates of the RNA-seq samples. Supplementary table S2. List of primers used in the qRT-PCR validation. Supplementary figure S1. Donut chart representing the Gene Ontology (GO) and KAAS (KEGG Automatic Annotation Server) functional annotations of all the transcripts pooled from the treatment (symbiotic nematodes) and control (axenic nematodes) groups. The top 10 enriched gene ontologies (GO) terms under each category of cellular component, biological process and molecular function (the inner circle) and top 10 ten enriched KEGG pathways are represented. The relative area under each GO term/KEGG ID indicates the number of transcripts mapping to that GO term/KEGG ID. Supplementary figure S2. A. Heat map showing the relative expression of transcripts in symbiotic and axenic early-adults of Heterorhabditis. The map was generated by using the FPKM values, and all the three replicates (R1-R3) are presented. B. Volcano plots representing differentially expressed transcripts (log2 fold change) in symbiotic nematodes as compared to axenic nematodes. Out of 754 differentially expressed transcripts, 547 transcripts were down-regulated, whereas, 207 transcripts were up-regulated in symbiotic nematodes. Supplementary figure S3. Bioinformatic analysis pipeline used to analyse the RNA-Seq data. [file 12864_2022_8952_MOESM1_ESM.docx]
